# High-throughput sequencing of sorted expression libraries reveals inhibitors of bacterial cell division

**DOI:** 10.1186/s12864-018-5187-7

**Published:** 2018-10-29

**Authors:** Daniel G. Mediati, Catherine M. Burke, Shirin Ansari, Elizabeth J. Harry, Iain G. Duggin

**Affiliations:** The ithree institute, University of Technology Sydney, Ultimo, NSW 2007 Australia

**Keywords:** Cell division, Uropathogenic *E. coli*, High-throughput, Genetic screen, DNA sequencing, Filamentation

## Abstract

**Background:**

Bacterial filamentation occurs when rod-shaped bacteria grow without dividing. To identify genetically encoded inhibitors of division that promote filamentation, we used cell sorting flow cytometry to enrich filamentous clones from an inducible expression library, and then identified the cloned DNA with high-throughput DNA sequencing. We applied the method to an expression library made from fragmented genomic DNA of uropathogenic *E. coli* UTI89, which undergoes extensive reversible filamentation in urinary tract infections and might encode additional regulators of division.

**Results:**

We identified 55 genomic regions that reproducibly caused filamentation when expressed from the plasmid vector, and then further localized the cause of filamentation in several of these to specific genes or sub-fragments. Many of the identified genomic fragments encode genes that are known to participate in cell division or its regulation, and others may play previously-unknown roles. Some of the prophage genes identified were previously implicated in cell division arrest. A number of the other fragments encoded potential short transcripts or peptides.

**Conclusions:**

The results provided evidence of potential new links between cell division and distinct cellular processes including central carbon metabolism and gene regulation. Candidate regulators of the UTI-associated filamentation response or others were identified amongst the results. In addition, some genomic fragments that caused filamentation may not have evolved to control cell division, but may have applications as artificial inhibitors. Our approach offers the opportunity to carry out in depth surveys of diverse DNA libraries to identify new genes or sequences encoding the capacity to inhibit division and cause filamentation.

**Electronic supplementary material:**

The online version of this article (10.1186/s12864-018-5187-7) contains supplementary material, which is available to authorized users.

## Background

Bacterial cell division genes were first discovered in filamentous, temperature-sensitive (*fts*) mutants [1]. Over 50 years later, it is expected that many more genes involved in the mechanism and regulation of division remain to be discovered [2, 3]. In addition to identifying these, it is of great interest to identify inhibitors of cell division, as potential anti-microbial agents or research tools [4, 5]. Filamentation is also observed in nature as a conditional response. For example, in uropathogenic *Escherichia coli* (UPEC), extensive, reversible filamentation occurs during the late stage of bladder cell infection in a UTI [6].

Bacterial cell division starts with the identification of an appropriate mid-cell site and the assembly of division proteins into a ring-shaped structure around the cell, at the inner surface of the cell envelope. This develops into a structure known as the divisome, which constricts the cell and builds new cell poles for the two daughter cells [3]. Most of the known regulators act at the initial assembly stage, although subsequent cell constriction and closure by the mature divisome must also be coordinated with chromosome replication and segregation.

In the *E. coli* cell cycle, known regulators of division include the nucleoid occlusion, Min, and Ter-linkage systems [7]. Nucleoid occlusion inhibits cell division in areas where the bacterial chromosomes (nucleoids) are located. This involves the binding of the SlmA protein to specific sites in the chromosome, where it inhibits polymerisation and assembly of the tubulin-like division protein, FtsZ [8]. The MinCD proteins concentrate on the inner membrane towards the poles of the cell, inhibiting divisome assembly there [9]. Overproduction of SlmA-DNA or MinCD results in filamentation [8, 10]. The Ter-linkage system provides positive regulation of division. Interactions between MatP, bound specifically to sites in the chromosome replication terminus region, and the early-stage division protein ZapB, promote divisome assembly at mid-cell [11]. This appears to help coordinate chromosome segregation and cell division [11, 12]. It has been recognised that as yet unknown cell cycle regulators of division are also likely to be present in *E. coli* [11, 13].

When compared to the processes involved in housekeeping cell cycle regulation of division described above, fewer regulatory pathways that lead to arrest of cell division and filamentation under specific conditions have been characterised. The best-known case is the DNA damage (or “SOS”) response [14]. DNA damage is sensed by the RecA protein, which then triggers auto-degradation of the SOS repressor LexA, and expression of SOS genes. One of the SOS genes encodes the FtsZ assembly inhibitor, SulA. The resulting filamentation allows time for DNA repair; when the SOS response is switched back off, the filamentous cells can resume division to generate short rods with intact chromosomes [10].

Another important example of conditional filamentation is seen during urinary tract infections (UTI). During a late stage of the intracellular infection cycle, UPEC grow into extensive filaments that may be hundreds of microns long and erupt from the overwhelmed host cell [15]. UPEC filaments are thought to help avoid phagocytosis by macrophages during infection and may improve bacterial dispersal and surface adhesion [16]. It has been suggested that UPEC filamentation in UTI occurs via a SulA-dependent pathway [17]. However, extensive SulA-independent filamentation by UPEC in experimental infection was recently observed, which instead required a known cell division protein, DamX [18]. DamX normally contributes to cell envelope synthesis during division [19], and was previously noted to cause extensive filamentation when overproduced [20, 21]. The exact roles of these proteins in UPEC need clarification, and the signalling pathway(s) that lead to filamentation during UTI remain to be identified.

Here, we report the development and utilization of a high-throughput DNA sequencing approach to identify genes, or genomic DNA fragments, that result in filamentation after induced over-expression from a plasmid-based DNA library. We applied this method to a UPEC strain capable of UTI-associated filamentation. This identified new and known inhibitors or mediators of *E. coli* cell division as well as potential components of the UTI-associated or other filamentation responses.

## Methods

### Bacterial strains, plasmids and growth conditions

*E. coli* BW25113 and JW0941–1 [22] were obtained from the *E. coli* Genetic Stock Center (Yale University, USA). *E. coli* UTI89 [23] and plasmids pBAD24 [24] and pLau80 [25] were kindly provided by J. Moller-Jensen. Strains were routinely cultured at 37 °C on solid or in liquid Luria Broth (LB), with 1% (*w*/*v*) NaCl.

For the cloning of individual ORFs, DNA fragments containing *fsaA, ybiY, aceE, pdhR, sdaA, lipA, pptE* and *pflC* were amplified from *E. coli* UTI89 genomic DNA (gDNA) with Phusion DNA polymerase (New England Biolabs - NEB). DNA oligonucleotide sequences and the integrated restriction sites are shown in Additional file 1: Table S2. The products were purified using the Isolate II PCR and gel-extraction kit (Bioline) before digestion and cloning into pBAD24, utilizing the restriction enzymes indicated in Additional file 1: Table S2. The ligation mixtures were transformed into electro-competent *E. coli* BW25113 cells, and clones selected on solid LB agar supplemented with ampicillin (100 μg/ml). Ampicillin resistant colonies were selected, the expected plasmid inserts were confirmed by Sanger single-read sequencing (Australian Genome Research Facility).

### Construction of the shotgun UTI89 genomic DNA expression library

Purified genomic DNA from *E. coli* UTI89 was partially digested with FatI (New England Biolabs), a 4-bp-recognition endonuclease that includes the ATG start codon, which would increase the frequency of in-frame ORF ligation to the expression vector compared to other 4-bp-recognition endonucleases. The gDNA fragments (1–5 kb range) were gel purified and ligated to pBAD24, which had been prepared by digestion with NcoI (NEB), dephosphorylated with Antarctic Phosphatase (NEB) and verified not to self-ligate by transformation of a vector-only ligation mixture. The library ligation mixture was used to transform *E. coli* JW0941–1 (*ΔsulA*) by electroporation, and clones were selected on LB agar supplemented with 100 μg/ml Ampicillin. Colonies arising after ~ 18 h growth at 37 °C were counted and then pooled by suspension and mixing in LB + 16% (*v*/*v*) glycerol, and stored in aliquots at − 80 °C.

### Cell sorting for enrichment of filamentous clones from the expression library

Mid-log cultures of *E. coli* JW0941–1/pBAD24 (vector only), JW0941–1/pLau80 (encoding FtsZ-YFP under P_BAD_ control) and the UTI89 gDNA expression library were diluted to A_600_ = 0.05 and induced with 0.2% (*w*/*v*) L-arabinose. Incubation was continued until A_600_ = 0.80. Culture samples were placed on ice and then analysed and sorted with an Aria II flow cytometer (BD Biosciences), as described previously [20]. Specifically, 500,000 events from the “filamentous” gate were initially collected (“yield sort”), and then the sample was re-sorted to obtain ~ 20,000 events of higher purity (“purity sort”). Cultures were analysed at an average of 37,000 events per second for the initial yield sort, and at 14,000 events per second for the purity sort. Purity sorted cells and 1 mL of the unsorted sample were used to inoculate 5 mL LB media containing ampicillin and 0.2% (*w*/*v*) D-glucose and grown overnight at 37 °C with shaking (~ 150 rpm). Plasmid DNA from both cultures was extracted and purified using the Isolate II Plasmid Mini Kit (Bioline).

### High-throughput multiplex DNA sequencing and data analysis

Plasmid DNA (5 ng) was prepared for sequencing using the Nextera DNA Library Prep Kit (Illumina), according to the manufacturer’s instructions. After the Nextera tagmentation and PCR amplification (tagging and barcoding) procedures, the DNA fragments were quantified on an Agilent Bioanalyzer and then underwent SPRI-select magnetic bead clean-up (Beckman-Coulter) at 0.8–0.5x left and right ratios (to select DNA of ~ 150–800 bp), according to the manufacturer’s instructions. The sorted and unsorted libraries were pooled, and quantified with an Agilent Bioanalyzer. The DNA was then diluted to 20 pM, including 5% PhiX genomic adapter-ligated DNA control library (Illumina). Sequencing was performed with an Illumina V3 150-cycle paired-end (2 × 75 cycles) flow cell and MiSeq instrument, according to the manufacturer’s instructions.

Sequence reads from the primary FASTQ files were demultiplexed according to their sample barcodes and then underwent quality control analysis and filtering using the FastQC software tool (Andrews, S. 2010), version 0.11.2. (http://www.bioinformatics.babraham.ac.uk/projects/fastqc/). Sequence read mapping to the UTI89 genome (Genbank NC_007946.1) [26] was done using Bowtie2 [27]. Reads were first strictly mapped (zero mismatches allowed) to the pBAD24 plasmid. Unaligned reads were then strictly mapped to the plasmid pUTI89. Finally, the unaligned reads remaining were mapped strictly to the chromosome of UTI89. Statistics of sequencing reads and the data in Table 1 were generated by using the SAMtools software package and Flagstat command [28]. The frequency of mapped reads and the analysis of data for Fig. 2 were determined with deepTools2 software [29]. Artemis [30] was used to visualize the alignments of the reference genome with the read data (BAM files) for inspecting hit regions and annotating the output from the peak detection (described below).

**Table 1:** Read frequencies from sequencing of plasmid libraries

Sample	No. of reads generated	No. of reads mapped to pBAD24 (%)	No. of reads mapped to UTI89 (%)
Filamentous-sorted 1	3,127,306	2,021,876 (64.6)	802,924 (25.7)
Reference-unsorted 1	2,093,673	1,414,249 (67.6)	470,937 (22.5)
Filamentous-sorted 2	4,462,451	2,279,041 (51.0)	1,223,720 (27.4)
Reference-unsorted 2	20,600,235	9,353,917 (45.4)	5,887,097 (28.6)

Genomic regions causing filamentation were identified by comparing the read depth of the filamentous-sorted sample compared to the read-depth of reference-unsorted sample with MACS (version 2.1.1) peak-detection software [31]. The alignment data of reads matched to the UTI89 genome were loaded into MACS using the Galaxy interface environment [32]. The paired-end data for each sample were then combined, and the filamentous-sorted data were input into MACS as the test/tag file and the reference-unsorted data as the control file. Sequence reads of 75 bp and genome length of 5.06 Mbp were specified. The *P*-value threshold cutoff for peak detection and the bandwidth parameter were the default values of 0.00005 and 300, respectively. The ranges for calculating regional lambda were defined as 1000, 5000 and 10,000 bp in the control file. The value *d*, as determined by MACS, was 200 for both replicate screens.

### Expression of cloned ORFs and analysis of cell phenotypes

Single colonies of BW25113-based strains, containing the cloned ORF’s in pBAD24, were used to inoculate liquid LB medium supplemented with ampicillin (100 μg/ml), and incubated overnight at 37 °C (~ 150 rpm). Cultures were then diluted in M9 minimal-media (supplemented with 0.4% (*v*/*v*) glycerol and 0.1% (*w*/*v*) casamino acids) to A_600_ = 0.02 and induced with the addition of 0.2% L-arabinose, and repressed with the addition of 0.2% (*w*/*v*) glucose, respectively. Cultures at A_600_ = 0.8 were fixed with volume of 2% (*w/v*) formaldehyde (prepared from a paraformaldehyde stock) and stored at 4 °C. Cell volume distributions were determined using a Multisizer 4 Coulter counter/cytometer (Beckman-Coulter), with a 50-μm aperture tube and Isoflow electrolyte (Beckman-Coulter) and calibrated with 2-μm latex beads. For microscopy, the cells were stained with FM4–64 (12.5 μg/mL) and Hoechst 33342 (0.5 μg/mL) and were then mounted on a 1% (*w/v*) agarose gel pad (in phosphate-buffered saline) and then imaged in phase-contrast and epifluorescence with a Zeiss Axioplan 2 microscope, with a 1.4 NA objective lens.

### Amplification and sub-cloning of fragments from identified genomic regions

The two loci, *ybiY-fsaA-moeB-moeA* and *ybeM-tatE-lipA* were first amplified from UTI89 gDNA using Phusion High-Fidelity DNA polymerase (NEB). The amplicons were purified and then partially digested with FatI (NEB). This DNA was ligated to the compatible NcoI site of pBAD24 using T4 DNA ligase. The ligation mixture was transformed by electroporation into *E. coli* BW25113 and clones selected on solid LB agar supplemented with 100 μg/ml ampicillin. Forty-eight ampicillin resistant colonies were picked into 96-well plates containing LB or M9 media with 100 μg/ml ampicillin and 0.2% (*w*/*v*) L-arabinose. BW25113 containing pBAD24 only was also used as a control strain. Plates were incubated for 7 h at 37 °C (~ 150 rpm) and cells adhered to 15-well poly-L-lysine-coated glass slides and examined by phase-contrast microscopy.

## Results

### A high-throughput screen based on flow cytometric sorting of DNA libraries

In order to identify genes or genomic regions that contain the potential to induce filamentation, a plasmid-based expression library of UPEC genomic DNA fragments was first constructed. Genomic DNA fragments of 1–5 kb from UPEC cystitis isolate UTI89 were ligated to the vector pBAD24, to place them under control of the arabinose-inducible promoter (P_BAD_). Approximately 35,000 colonies arising from transformation of *E. coli* JW0941–1 (K-12 background, *ΔsulA*) were resuspended and pooled for storage. The *ΔsulA* host strain was selected in order to identify mediators of filamentation that are independent of the well-characterised SulA division inhibitor.

**Fig. 1:**
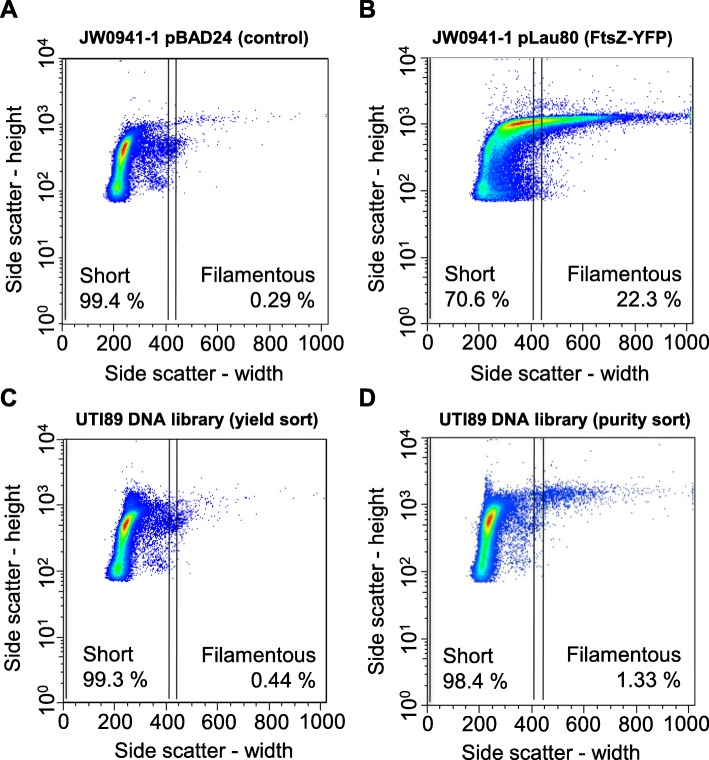
Analysis and purification of filamentous bacteria from a UPEC DNA-expression library by flow cytometry. Flow cytometry was carried out with the control populations of strains JW0941–1/pBAD24 (**a**) and JW0941–1/pLau80 (containing FtsZ-YFP, which induces strong filamentation) (**b**). The sorted populations of the UTI89 gDNA expression library in JW0941–1 are also shown: an initial high-speed, high-yield sort (**c**) and a subsequent lower speed “purity sort” to further enrich for filamentous cells (**d**). Dots represent single events plotted as side-scatter values of peak height and peak width, which correlates with filament length. Event density is colour coded with a heat-map of red (high density) to blue (displaying individual events). The gates for sorting are indicated with vertical lines, and the percentages of events contained within that gate are indicated. The “short” gate was determined to encompass greater than 99% of the control bacterial population (short cells), JW0941–1/pBAD24 (**a**)

Transcription from P_BAD_ was induced by the addition of 0.2% (*w*/*v*) L-arabinose to a mid-log phase culture of the library, for 4 generations. These conditions were chosen to allow strong induction of transcription from P_BAD_, such that moderate or strong inhibitors of division can function, whilst providing sufficient time for these clones to generate filaments of lengths that were amenable to purification by flow cytometry [20]. Flow cytometry cell sorting was then used to collect cells that had developed a filamentous morphology. Control cultures of JW0941–1/pBAD24 (“short” cells; Fig. 1a) and JW0941–1/pLau80 (“filamentous” cells, caused by over-production of FtsZ-YFP; Fig. 1b) were analysed too.

Approximately 20,000 cells that displayed a filamentous phenotype were isolated from the induced expression library (Fig. 1c and d, “filamentous” gate) on the basis of the side-scatter (pulse width) parameter, and they were then transferred to LB medium including glucose—to supress transcription from P_BAD_. These cells were then incubated overnight at 37 °C to allow reversal (division) of filaments and growth of viable bacteria (ongoing growth of filaments was expected to be eventually lethal). Plasmid DNA from this filamentous-sorted library was then purified and subjected to high-throughput sequencing, to identify the cloned UTI89 DNA that caused inducible filamentation. A reference sample from the same culture, treated in the same manner but not sorted, was also sequenced in the same multiplexed sequencing run. The whole experiment was performed in duplicate, starting with two frozen aliquots of the same library.

The mapped sequence-read frequencies for each sample showed that the expected majority of reads mapped to the vector pBAD24, with most of the remainder matching the UTI89 chromosome or native plasmid, pUTI89 (Table 1). The reads matching pBAD24 were excluded from further analysis. A plot of the reference library’s distribution of read depth per bp showed that the library covers 100% of the UTI89 chromosome by at least 1 read per bp, while < 1% was covered by at least 30 reads per bp (reference-unsorted 2 data, Additional file 2: Figure S1A).

**Fig. 2:**
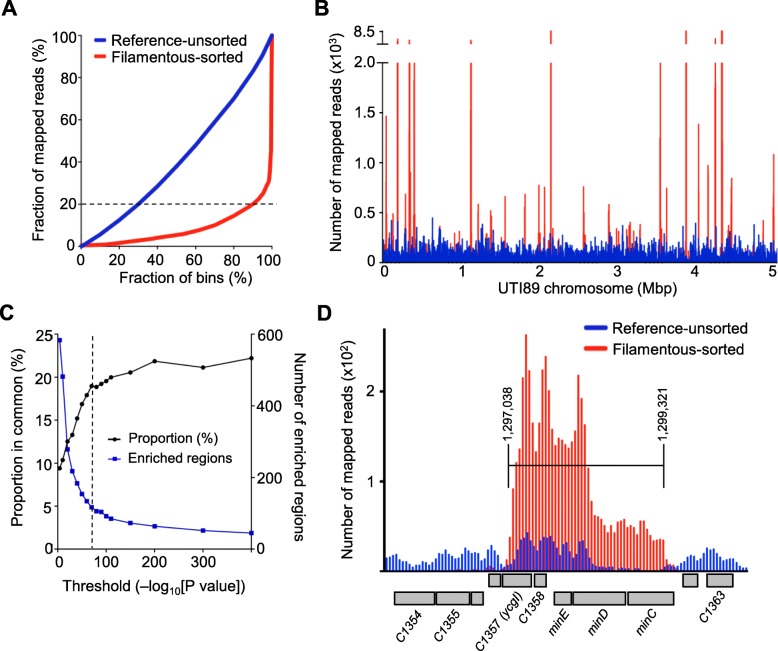
High-throughput DNA sequencing of plasmid libraries from the reference-unsorted and filamentous-sorted populations. **a** Comparison of the distribution of mapped reads in 10 kb UTI89 chromosomal bins, showing the degree of enrichment of mapped reads in specific bins after flow cytometry. The dotted line indicates 20% of the mapped reads (see text). **b** The distribution of read depth along the ~ 5.06 Mbp UTI89 chromosome in the filamentous-sorted sample (red) and the reference-unsorted sample (blue), with a bin size of 1 kb. **c** The proportion of enriched regions found in common in both biological replicates versus their corresponding threshold significance score (−log_10_[*P*-value]) (left-hand y-axis), and the total number of enriched regions from both replicates (right-hand *y*-axis). The score of 70 (dotted line) represents the selected threshold criteria for display in Table 2. **d** The number of reads mapped to the region 1,295,150 – 1,302,200 bp of UTI89 in the filamentous-sort (red) and the reference-unsort (blue) samples within a bin window of 50 bp. Large read depth numbers in the filamentous-sort sample (replicate 1, red) can be seen against the reference-unsorted sample in the region spanning from the *ycgI* homolog to the *minCDE* genes (the region identified by MACS is shown by the bracket). Open reading frames (grey boxes) encoded by the upper and lower strands are indicated by their relative positioning below the axis

We then detected genomic regions that were significantly enriched in reads from the filamentous-sorted samples, compared to the respective reference-unsorted samples. A global analysis of the degree of enrichment of specific genomic regions is shown in Fig. 2a; the filamentous-sorted sample showed a strong enrichment of reads in relatively fewer 10 kb genomic windows (or bins) compared to the reference-unsorted library. For example, 20% of the mapped reads from the filamentous-sorted sample were located in ~ 90% of the bins, while the remaining 80% of mapped reads were clustered in only 10% of the bins (dotted line, Fig. 2a). In contrast, 20% of mapped reads from the reference-unsorted sample were located in ~ 30% of bins, while the remaining 80% of mapped reads were distributed throughout ~ 70% of bins. This comparison demonstrates the effectiveness of the combination of cell sorting and high-throughput sequencing of plasmid DNA in enriching and identifying genomic regions. We characterised this enrichment further by plotting the number of mapped sequencing reads in 1 kb windows, to obtain an overview of the relative frequency of mapped reads along the entire chromosome. As may be seen in Fig. 2b, strong peaks of enrichment of read counts in the filamentous-sorted sample compared to the reference-unsorted sample were evident at loci distributed throughout the chromosome.

### Identifying genomic regions that encode mediators of bacterial filamentation

We identified the significantly enriched regions in the filamentous-sorted data by using the peak-detection software MACS [31]. The input data were the combined paired-end reads aligned to the UTI89 genome, from the filamentous-sorted and reference-unsorted samples. The aligned data (deposited in the European Nucleotide Archive under accession number PRJEB23331) were visualized with Artemis [30] to inspect regions identified by MACS. The complete annotated output of enriched regions from each replicate, and a list of the 55 regions that were found common to both replicate screens, are provided in Additional file 3: Table S1. We ranked these regions with respect to their significance score (−log_10_[*P*-value]), and found that the number of enriched regions identified by the two replicate screens corresponded strongly, above a significance score of ~ 70 (Additional file 2: Figure S1B). Furthermore, the fraction of enriched regions specifically in common between replicates was maintained near the maximum correspondence (~ 20%), at significance scores ≥70, but reduced markedly at lower significance scores (Fig. 2c). By applying this high-stringency significance threshold (≥70), 23 regions were identified in common between both replicate screens (Table 2). These regions are therefore most strongly implicated in causing filamentation.

**Table 2:** UTI89 chromosomal regions identified to contain mediators of filamentation (high stringency hits in both replicate screens)

Genomic Region (bp)^a^	Locus ID	Gene Name(s)^b^	COG^c^	Length (bp)	Fold Enrichment^d^	Significance score^d^
14,519–15,641	UTI89_C0017	*dnaJ*	O	1123	6.9	197.3
	UTI89_C0018	*gef (hok)*	R			
361,550–364,162	UTI89_C0348	*yahD*	R	2613	17.1	935.0
	UTI89_C0349	*yahE*	S			
	UTI89_C0350	*yahF*	C			
640,717–641,788	UTI89_C0629	*ybeM*	R	1072	4.3	112.5
	UTI89_C0630	*tatE*	U			
	UTI89_C0631	*lipA*	H			
690,222–691,856	UTI89_C0684	*glnS*	J	1635	79.2	8504.0
1,016,170–1,016,793	UTI89_C1021	*matP*	MD	624	9.1	575.5
	UTI89_C1022	*ompA*	M			
1,139,704–1,141,232	UTI89_C1147		S	1529	9.6	321.3
	UTI89_C1148	*(cbeA/yeeU)*	S			
	UTI89_C1149	*(cbtA/yeeV)*	S			
	UTI89_C1150		S			
1,245,504–1,247,434	UTI89_C1276	*bet*	S	1931	415.2	43,744.9
	UTI89_C1277	*gamW*	R			
	UTI89_C1278		S			
	UTI89_C1279	*kil1*	R			
	UTI89_C1280	*λ-cIII*	S			
	UTI89_C1281	*(ea10)*	S			
	UTI89_C1282	*ral*	R			
1,297,038–1,299,321	UTI89_C1357	*(ycgI)*	S	2284	4.7	174.7
	UTI89_C1358		S			
	UTI89_C1359	*minE*	D			
	UTI89_C1360	*minD*	D			
	UTI89_C1361	*minC*	D			
1,392,009–1,395,131	UTI89_C1459	*exoO*	SR	3123	70.7	10,266.0
	UTI89_C1460		S			
	UTI89_C1461	*dicB*	D			
	UTI89_C1462	*dicF*	RD			
	UTI89_C1463	*ydfC*	S			
	UTI89_C1464	*ydfB*	S			
	UTI89_C1465	*ydfA*	S			
	UTI89_C1466	*dicA*	KT			
	UTI89_C1467	*dicC*	R			
1,961,291–1,963,326	UTI89_C2055	*zwf*	G	2036	19.0	1044.3
2,011,010–2,012,850	UTI89_C2102	*(yecI/ftnB)*	P	1841	6.7	159.7
	UTI89_C2103	*yecJ*	S			
	UTI89_C2104		S			
	UTI89_C2105	*yecR*	SR			
	UTI89_C2106	*ftnA*	P			
2,632,799–2,635,972	RS13095		S	3174	15.0	1023.6
	UTI89_C2682	*λ-cIII*	S			
	UTI89_C2683	*kil2*	R			
	UTI89_C2684		S			
	UTI89_C2685		S			
	UTI89_C2686	*erf*	S			
	UTI89_C2687		S			
	UTI89_C2688	*hkaJ*	S			
	UTI89_C2689	*hkaH*	S			
	RS13145		S			
2,927,393–2,928,805	UTI89_C2990		G	1413	193.2	17,052.9
	UTI89_C2991		S			
	UTI89_C2992		S			
	UTI89_C2993		S			
2,933,266–2,935,042	UTI89_C3000	*ymfN*	K	1777	36.0	3042.5
	UTI89_C3001	*ymfM*	RD			
	UTI89_C3002	*ymfL*	S			
	UTI89_C3003	*ymfT*	S			
	UTI89_C3004	*ymfK*	KT			
3,029,776–3,031,529	UTI89_C3112	*nlpD*	M	1754	92.6	23,091.7
	UTI89_C3114	*pcm*	O			
3,162,699–3,166,774	UTI89_C3238	*tas*	C	4076	8.1	372.1
	UTI89_C3239	*lplT*	U			
	UTI89_C3240	*aas*	IQ			
3,426,907–3,428,106	UTI89_C3489	*glnE*	OT	1200	12.8	1200.0
	UTI89_C3490	*ygiF*	S			
3,553,759–3,555,227	UTI89_C3621	*ispB*	H	1469	11.8	666.4
	UTI89_C3622	*nlp*	K			
	UTI89_C3623	*murA*	M			
3,723,755–3,724,973	UTI89_C3832	*rpoB*	K	1219	42.4	7961.0
	UTI89_C3834	*rplL*	J			
	UTI89_C3835	*rplJ*	J			
3,767,241–3,770,673	UTI89_C3884	*rpe*	G	3433	61.3	5157.4
	UTI89_C3885	*dam*	L			
	UTI89_C3886	*damX*	D			
	UTI89_C3887	*aroB*	E			
3,921,231–3,922,700	UTI89_C4038	*hmuV*	P	1470	19.0	1402.3
	UTI89_C4039	*yhiD*	S			
4,049,515–4,052,967	UTI89_C4158	*yibP (envC)*	D	3453	51.7	12,724.5
	UTI89_C4159	*yibQ*	S			
	UTI89_C4160	*yibD*	M			
	UTI89_C4162	*tdh*	ER			
4,997,073–4,999,597	UTI89_C5098		S	2525	14.8	584.6
	UTI89_C5099		S			
	UTI89_C5100		S			
	UTI89_C5101	*yfdN2*	S			
	UTI89_C5102		S			

^a^Coordinates were determined from Genbank accession NC_007946.1 as the minimal region identified in common between both replicates, with an overlap > 100 bp

^b^Genes within or overlapping the hit region are shown, and named based on characterized homologs in other strains; alternative names may also be given in parentheses. Hypothetical or proteins with no characterized homologs are left blank

^c^*COG* Clusters of Orthologous Groups functional classification code

^d^The significance score (log_10_[P-value]) and fold-enrichment are shown as the means from the two replicate screens

The list in Table 2 includes regions that contain genes with a known involvement in cell division and its regulation, such as *zapB* [33] and *minCDE*. Figure 2d shows the data for the *minCDE* locus, encoding the well-known spatial regulator of division. The strongest region of relative enrichment coincides with the *minC* gene, which is the FtsZ inhibitor of the system [34]. The region of enrichment extended downstream of *minCDE*, including a conserved gene of unknown function (C1358), and a pseudogene C1357 (the homolog of *ycgI* in *E. coli* K-12), suggesting that these genes or fragments in this region might affect cell division too. Alternatively, they may be represented due to their linkage to *minCDE* in our library. Some of the strongest hits were located in prophages or prophage remnants that have previously-demonstrated roles in blocking cell division, including *kil1* and *kil2* (λ-prophages), *dicB/dicF* (Qin prophage), and the *ymfN-ymfK* region of the CP-933O (e14) prophage. There were also numerous genes encoding membrane-associated proteins, such as *ompA* and *rfaG* (*waaG*), and others involved in peptidoglycan cell wall structure, including *nlpD* (Additional file 3: Table S1B). We also identified a region containing *damX*, which plays a role in cell division [35] and was previously shown to inhibit cell division when overexpressed, causing a filamentous phenotype [21]. DamX has recently been specifically implicated in the conditional filamentation seen during urinary tract infections [18].

Amongst the expanded list of 55 identified regions that were common to both screens at the default threshold (Additional file 3: Table S1B) were other genes with a known involvement in cell division, such as *zapA* [36], suggesting that this list also contains significant inducers of filamentation. We expect that some regions only identified with strong significance in one of the screens may also contain genuine inducers of filamentation (Additional file 3: Table S1A), but they might have been missed in one of the screens due to limited sampling of cells from the library by the flow cytometry. Other regions encoding a capacity to cause filamentation may not have been cloned in the correct position with respect to P_BAD_ in the library and were therefore not detected in either screen.

### Identification and verification of fragments that cause filamentation

**Fig. 3:**
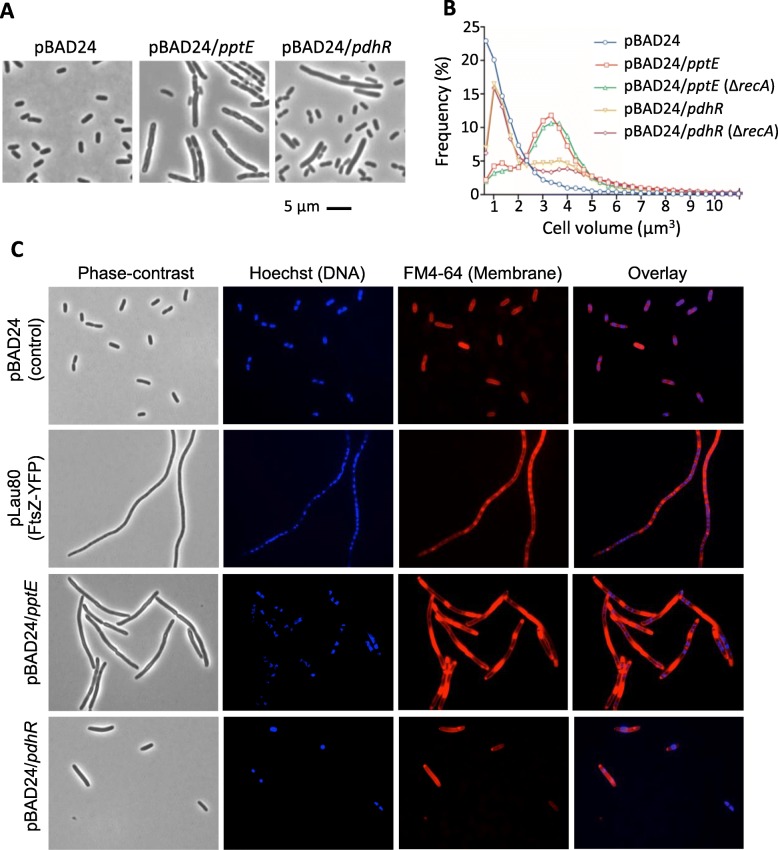
Overexpression of the *pptE* and *pdhR* ORFs cause *E. coli* filamentation independent of *recA* (SOS response). The *pptE* and *pdhR* ORFs from UTI89 were cloned into pBAD24, transformed into BW25113, and BW25113 (Δ*recA*), and cultures induced with 0.2% L-arabinose in M9 medium. Fixed cells were analysed by microscopy (**a**) and Coulter cytometry to obtain cell volume distributions (**b**). **c** Fixed cells were stained using the Hoechst 33342 (DNA) and FM4–64 (membrane) and imaged by fluorescence microscopy. An overlay of the Hoechst and FM4–64 channels is also shown, indicating the obvious anti-correlation between the two stains, suggesting a possible physical exclusion of these features

To investigate whether particular open reading frames (ORFs) from the identified DNA regions were responsible for the observed filamentation, we initially selected eight candidate ORFs from six of the hit regions of differing statistical strength, including some that were only observed in one screen and others that overlap the edge of a hit region (Additional file 3: Table S1A). These ORFs were individually cloned into pBAD24: *fsaA, ybiY, aceE, pdhR, sdaA, lipA, pptE* and *pflC*. Two of the cloned ORFs produced a reproducibly elongated or filamentous phenotype after the controlled induction period, namely *pptE* and *pdhR*, which showed a mean cell volume of 3.6 μm^3^ and 2.8 μm^3^, respectively, compared to 1.4 μm^3^ for the strain containing the vector-only (BW25113/pBAD24) (Fig. 3).

As may be seen in Fig. 3a and b, the cell size distributions of cultures overexpressing *pptE* and *pdhR* differed. Overexpression of *pdhR*, encoding the pyruvate dehydrogenase operon repressor, caused a mixed population of short cells and filaments. By comparison, overexpression of *pptE*, encoding a putative phosphoenolpyruvate-protein phosphotransferase, caused a much higher proportion of the cells to form filaments. Both genes caused their same characteristic filamentation in the absence of the SOS response regulator *recA* (Fig. 3b). Strikingly, staining of the membrane (FM4–64) and DNA (Hoechst 33342) in the *pptE* overexpression strain revealed irregularly positioned nucleoids in the filaments, and discontinuous zones of strong FM4–64 staining that corresponded to apparent inclusions in phase-contrast images (Fig. 3c). Similar inclusions were also present during conditions expected to result in significantly lower *pptE* expression (0.02% L-arabinose), although these conditions did not cause significant filamentation (Additional file 4: Figure S2). These observations suggest that the inclusions may be lipid-rich, and that filamentation occurs in this strain as a secondary effect, requiring higher levels of expression.

**Fig. 4:**
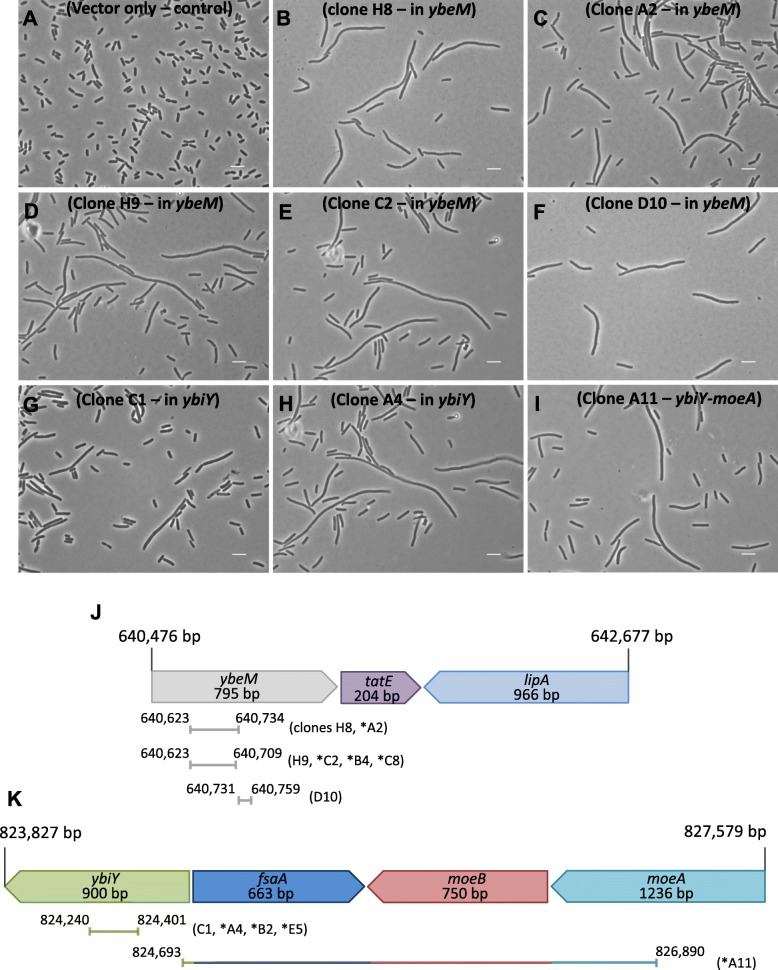
Partial fragments of ORFs cause filamentation. FatI fragment sub-clones from the *ybiY-fsaA-moeB-moeA* and *ybeM-tatE-lipA* regions were screened (48 colonies each) for filamentation after growth and induction for 7 h at 37 °C. The indicated clones were identified as causing filamentation. **a** BW25113/pBAD24 control, (**b**) Clone H8 (*ybeM*), (**c**) Clone A2 (*ybeM*), (**d**) Clone H9 (*ybeM*), (**e**) Clone C2 (*ybeM*), (**f**) Clone D10 (*ybeM*), (**g**) Clone C1 (*ybiY*), (**h**) Clone A4 (*ybiY*), (**i**) Clone A11 (*ybiY-moeA*). **j** The *ybeM-tatE-lipA* region and sub-clones identified as capable of causing filamentation; three fragments throughout the 7 clones, of 112 bp, 87 bp and 29 bp were identified within the *ybeM* gene. **k** Map of fragments within the *ybiY-fsaA-moeB-moeA* locus capable of causing filamentation. Some of the fragments indicated in (**j**) and (**k**) were isolated in multiple clones (see Table 3).indicated in parentheses). The asterisk indicates that the expressed fragments in these clones were in the opposite orientation in pBAD24 relative to the expected wild-type transcription of the indicated genes in UTI89. [The A11* clone was orientated with respect to pBAD in the order *ybiY* (partial) - *fsaA* - *moeB* – *moeA* (partial)]

The six tested ORFs that did not show an obvious filamentous phenotype had average cell volumes of 1.3–1.6 μm^3^ (Additional file 5: Figure S3A). We considered it likely that other ORFs, a combination of multiple ORFs, a sub-fragment of an ORF or an expressed intragenic region might have caused the observed enrichment in the screens. We therefore further investigated two of the hit regions in which the preliminary whole-ORF testing above did not reveal filamentation, i.e. *ybeM-tatE-lipA* (2186 bp) and *ybiY-fsaA-moeB-moeA* (3754 bp). Sub-libraries of the two PCR-amplified regions were created by cloning FatI partial-digest products into pBAD24, and 48 transformants from each were cultured individually in M9 medium with L-arabinose for 7 h. Microscopy revealed a total of 12 clones from the two sets as filamentous, and the cloned fragments from these were sequenced (Fig. 4).

Seven of the filamentous clones contained fragments from within the *ybeM* ORF that were out-of-frame with respect to P_BAD_ in the library. Two overlapping fragments of 112 bp and 87 bp were found in six of these clones (Fig. 4j); interestingly, both orientations of these fragments were observed amongst the clones, suggesting that two different products could trigger filamentation in this fragment. Alternatively, the region could conceivably contain its own Ara-inducible division inhibitor. The remaining clone (D10) contained a downstream fragment of only 29 bp within *ybeM* (encoding an amino acid sequence MFLQRRGAH).

From the *ybiY-fsaA-moeB-moeA* region, four of the five sub-clones identified as filamentous contained a 161 bp fragment in either orientation from within the *ybiY* ORF, despite the previous observation that the full-length *ybiY* ORF did not cause filamentation (Additional file 5: Figure S3A). Also, a larger downstream region of 2197 bp was identified (clone A11). This region spanned across the majority of the *ybiY-fsaA-moeB-moeA* region (Fig. 4k), with transcription in pBAD24 expected to start from within *ybiY* in the reverse orientation with respect to the expected transcription of *ybiY* in its genomic context. These findings show that sub-ORF in-frame fragments (e.g. clone C1 from *ybiY*) and out-of-frame fragments (e.g. clones H8, H9 and D10 from *ybeM*) can induce strong filamentation, even though the full-length ORFs (*ybiY* and *ybeM*) did not (Additional file 5: Figure S3B), suggesting that these fragments inhibit cell division via a mechanism that did not evolve for such a function in the whole ORFs they are located in.

## Discussion

We have developed a widely applicable high-throughput screen, combining flow cytometry cell sorting with high-throughput sequencing of a plasmid-based DNA expression library, to identify genes or genomic DNA fragments that act as inducible inhibitors of bacterial cell division and lead to bacterial filamentation. In this study, the filamentous clones isolated after expression of the genomic fragment library were recovered and amplified by overnight growth under conditions that supress expression. Since unlimited growth of bacterial filaments is expected to result in lysis, the recovery period was expected to enrich for reversible inhibitors of division, which were considered likely to be more physiologically relevant. We applied the method to *E. coli* UTI89, and identified 55 genomic regions that were enriched in the replicate screens. The identified genomic regions included numerous genes with known roles in cell division and its regulation, confirming that the method successfully identifies genes of biological relevance. Several previously uncharacterised genes were also identified that may represent new inhibitors or mediators of division, or be part of the UTI-associated or other filamentation responses. In addition, we identified a number of short predicted transcripts or peptides that are not predicted to be expressed from their genomic location in wild-type cells but were detected as sub-ORF, out-of-frame, or intergenic fragments. One fragment of only 29 bp, encoding a potential peptide (MFLQRRGAH), acts as an inhibitor of division in our screens. These or other similarly identified sequences could represent novel peptide or small RNA inhibitors of bacterial division. It is therefore expected that expanded screens of large and diverse DNA libraries could reveal other new inhibitors with potential applications.

An expectation of using a genomic fragment library for the screens described here is that not all genes will be appropriately positioned to be expressed from the plasmid. Furthermore, the size of our library (~ 35,000 clones) may also somewhat limit the screens, and they are therefore not considered fully comprehensive. Consistent with this, we identified six out of the 12 genomic regions previously identified by Sanger sequencing of clones obtained after a similar flow-cytometry screen was performed with *E. coli* K-12 (DH5α) [20]. The genomic regions in common with this study contained genes such as *damX* (that has been implicated in the UTI-filamentation response pathway [18]), *rplL-rpoB*, and two regions of prophage elements e14 (*ymfL*/*ymfM*) and Rac (*kil*). Another possible limitation relates to the yields obtained from the cell sorter flow cytometer (~ 20,000 cells per screen). Consistent with such a sampling limitation, a number of enriched regions were detected with statistical significance in only one of the two screens (Fig. 2c and Additional file 3: Table S1). Genes from some of these regions play known roles in cell division (e.g. *dedD*). We therefore recommend that other such regions are also considered as potential candidates for verification. These data may be directly assessed by referring to Additional file 3: Table S1A and the genomic alignments available from the European Nucleotide Archive (accession number PRJEB23331).

Given our identification of both physiological and likely non-physiological classes of genomic fragments that lead to filamentation, we suggest that further screens aiming to focus on physiologically-relevant gene identification could utilize comprehensive complementary DNA (cDNA) expression libraries, whereas others focusing on artificial peptide inhibitors could use diverse small fragment libraries (e.g. from environmental or randomized synthetic DNA sources). The cDNA screen results should still be followed up by determining whether the identified ORFs relate to a specific pathway in controlling wild-type cell division, or whether they cause filamentation via an artefactual consequence of overexpression (e.g. via substantive changes to envelope physiology that secondarily inhibits normal cell division). Both approaches would benefit from the development of the flow cytometry or other methods to provide a significantly greater yield of filamentous bacteria from the expression library.

An intriguing aspect of our screen results was the presence of genes within the regions causing inducible filamentation that have known roles in central carbon metabolism, including gluconeogenesis (*zwf*) (Table 2 and Additional file 3: Table S1). Glucose metabolism has previously been implicated in inhibiting cell division in *Bacillus subtilis*, via the glucosyltransferase, *ugtP*, which acts on FtsZ to delay cell division in the presence of UDP-glucose [37]. A similar UDP-glucose-dependent mechanism has also been found in *E. coli* with the functional analog, *opgH* [38]. Interestingly, our screens revealed a region containing a fragment of *rssB* and the whole downstream *galU* gene (Additional file 3: Table S1). GalU catalyzes the synthesis of UDP-glucose, which could conceivably stimulate the division-inhibition mediated by OpgH. However, previous overproduction of a GalU fusion protein did not cause filamentation [38]. Preliminary hits from our screen such as the *rssB*-*galU* region need to be checked by defining the specific regions and conditions that cause the filamentation.

We chose several genomic regions for following up and verifying the results of the screens, some of which encoded potentially novel division-metabolism links. Of particular note was the identification of a genomic region in one of our replicate screens containing the *pdhR* and *aceE* genes, encoding the pyruvate dehydrogenase complex regulator and pyruvate dehydrogenase e1 subunit, respectively. We showed that over-expression of the PdhR ORF caused filamentation, independent of the SOS pathway (*recA*) (Fig. 3)*.* The PdhR regulator is controlled by the concentration of pyruvate in the cell. When ample pyruvate is present, PdhR does not repress its target genes (including its own PDH operon), whereas in low-pyruvate conditions, PdhR specifically binds DNA and represses expression of numerous metabolic genes and the cell division gene cluster, *dcw*, which includes *ftsZ* [39]. In this way, low pyruvate (nutrient poor) conditions might reduce the frequency of division, coupling it to the reduced cell growth rate. Our finding that *pdhR* overexpression inhibits division therefore lends support to this notion. In *B. subtilis*, pyruvate metabolism also plays an important role in regulating division; pyruvate dehydrogenase was found to affect the assembly of the FtsZ-ring in a pyruvate-dependent manner [40]. It was suggested that this positively regulates FtsZ assembly and division in nutrient-rich conditions to appropriately couple division with growth rate. Therefore, this important final stage of glycolysis appears to be a nexus in the coupling of cell growth and division in these two highly divergent bacteria, but the underlying mechanisms that evolved to establish this regulation appear to be very different. A similar occurrence of convergent evolution was noted for the UDP-glucose-dependent mechanisms of division control, mentioned above [38].

The second whole-ORF that we discovered to encode a strong inducer of filamentation was *pptE* (Fig. 3). PptE is uncharacterised in UTI89, but is a putative phosphoenol-pyruvate-protein phosphotransferase, that is present in extraintestinal pathogenic *E. coli* (ExPEC) strains (phylogenetic group B2) including UPEC, avian pathogenic *E. coli* (APEC) and neonatal meningitis *E. coli* (NMEC). The *pptE* gene varies in size substantially amongst *E. coli* strains. A smaller remnant of *pptE* (0.34 kb out of the total 2.38 kb ORF) is evident across some UPEC strains [41], although UTI89 has the full length ORF. Overexpression of *pptE* led to major cell structure and morphology effects in *E. coli* (Fig. 3, Additional file 4: Figure S2). It is possible that *pptE* plays an additional uncharacterised role in UTI89 cell division or structure, but an understanding of its normal physiological role(s) awaits further characterization. The overexpression phenotypes of *pptE* and other genes identified here offer new leads towards defining their roles.

Some of the strongest and most frequent inducers of filamentation from our screens were regions that contained specific prophage genes, several of which have been previously studied as strong inhibitors of *E. coli* cell division. For example, the *kil1* gene in a λ-prophage was identified in our screens, and this gene encodes a known inhibitor of cell division that acts on FtsZ assembly [42]. Other prophage regions in the list include a large region of the Qin prophage, containing known division inhibitors *dicB* and *dicF*. The gene *dicB* encodes an activator of the endogenous *minC* division regulator/inhibitor, whereas *dicF* is a small RNA inhibitor of *ftsZ* gene expression [43, 44]. The *ymfN-ymfK* region of the e14 prophage was identified (Table 2), which was previously reported to contain an SOS-inducible cell division inhibitor, originally termed *sfiC* [45]. Furthermore, known prophage toxins from toxin-antitoxin pairs affecting cell division, including *cbtA* (*yeeV*) and *gef* (*hok*) [46, 47], were identified. These common functions of the prophage-associated genes therefore appear to reflect a wide-spread bacteriophage strategy to block cell division during lysis, which likely aids bacteriophage proliferation or dissemination. At least some of these genes in remnant or defective prophages appear to have been co-opted by the host bacteria for regulatory purposes in stress responses [48, 49].

We applied our method to *E. coli* UTI89, as it might contain specific regulators of the UPEC filamentation response seen in UTIs that are not present in other strains of *E. coli*. Alternatively, the filamentation response in UTI may involve a more general response pathway that is activated strongly under the conditions bacteria experience during UTI, such as the SOS response that was first postulated for UTI-associated filamentation [17]. We identified seven genomic regions in the reproducibly filamentous expression clones that did not contain direct homologs in *E. coli* K-12. These included regions containing prophage genes that are implicated in inhibition of cell division (Additional file 3: Table S1B), and some of these might have been co-opted by cells for regulatory purposes [48]. At present it is not clear whether these or other more wide-spread genes are involved in the UPEC filamentation response. However, the relevance of our screen towards the goal of identifying the components that are potentially involved in the UPEC filamentation response is exemplified by our identification of *damX*, which was previously identified amongst a set of genes that were upregulated during the filamentation stage of infection [18]. DamX has a known involvement in division, and its overexpression causes filamentation, particularly when the bacteria are surface bound [18, 21]. DamX was essential for the UPEC filamentation response observed during infection [18].

Other components of the regulatory pathway that leads to UPEC filamentation (via DamX) are still unknown. Interestingly, amongst our list of 55 UTI89 genomic regions causing inducible filamentation (Additional file 3: Table S1B), there were 16 ORFs that showed elevated expression during the filamentation phase of bladder cell infection [18]. These genes encode the outer membrane protein, OmpA (which is also under strong positive selection in UPEC strains [26]) and other proteins involved in peptidoglycan cell wall structure (NlpD and PbpG)*.* Several relatively poorly characterized genes, such as *ygaV*, were also detected in both studies. YgaV is a predicted transcriptional regulator that is part of a two-component regulator (YgaVP) responsive to tributyltin exposure, but is of unknown function [50]. These genes were therefore recognized as candidates that might act as regulators in the UTI-filamentation response.

## Conclusions

A high-throughput sequencing-based method for genome-wide identification of genes and DNA fragments that encode a capacity to induce filamentation in *E. coli* was successfully developed and applied. This revealed genes from several prophages, as well as loci that have known or novel roles in bacterial cell division or filamentation, and has helped refine a list of candidate genes that may be involved in the UPEC filamentation response pathway seen extensively in UTIs. Several short DNA fragments that cause filamentation were also identified and these may have potential uses as inhibitors of cell division. Our approach can be used to carry out comprehensive surveys of diverse DNA libraries, to identify new sequences encoding the capacity to arrest cell division and induce filamentation.

## Additional files

Additional file 1: **Table S2.** Primer sequences used for cloning the indicated ORFs into pBAD24. PCR products generated using these primers in the appropriate combinations were digested with the indicated restriction enzymes as appropriate for cloning. (XLSX 9 kb)

Additional file 2: **Figure S1.** Additional analyses of DNA sequencing data. (A) Plot showing the global read coverage of the UTI89 chromosome of the UTI89 genomic library, based on the read data from the “reference-unsorted 2” sample (Table 1). The library effectively covers the complete genome of UTI89, at a depth of 1–30 reads per bp. (B) A comparison of the number of identified enriched regions in replicate screens 1 and 2 by the MACS peak detection software (see Materials and Methods). The number of identified enriched regions were ranked by their significance score (−log10[*P*-value]), and the significance scores plotted against raw numbers of the identified regions. The dotted line represents the threshold of ≥70 P-value as the high-stringency criterion used to generate Table 2. (PDF 176 kb)

Additional file 3: **Table S1.** *E. coli* UTI-89 genomic regions identified in overexpression screens for mediators of filamentation. Table S1A contains the combined MACS output from the two replicate screens. Regions indicated in bold text represent those found in common between the replicates (with an overlap of 100 bp or more). Those underlined indicate the 23 high-stringency regions found in common between replicates (with an overlap of 100 bp or more) and also were above the P-value threshold of 70 (see also Table 2). The gene name(s) included were determined by annotating the region from start to end (bp) and listing all ORFs within or overlapping those coordinates. This therefore will include partial ORFs that may not directly cause filamentation; further mapping may be required in these cases. Table S1B shows only the regions identified in common between the two replicate screens (with overlap of 100 bp or greater), and identifies ORFs within these that were not found to have direct homologs in *E. coli* K-12. (XLSX 69 kb)

Additional file 4: **Figure S2.** *pptE* over-expression causes major effects on *E. coli* cell structure and morphology. BW25113 + pBAD24/*pptE* was grown in M9 minimal medium and induced with L-arabinose at the indicated concentrations and then fixed and stained with Hoechst 33342 (DNA) and FM4–64 (membrane) stains and then visualised by fluorescence microscopy. (A) 0.2% L-arabinose. (B) 0.02% L-arabinose. The intracellular inclusion-like structures and FM4–64 staining abnormalities were observed at both induction concentrations, whereas significant filamentation was only observed at the higher L-arabinose concentration. (PDF 1184 kb)

Additional file 5: **Figure S3.** ORFs *fsaA, ybiY, aceE, lipA, sdaA,* and *pflC* do not cause substantial filamentation when overexpressed. The ORFs were expressed from pBAD24 in BW25113 by induction with 0.2% L-arabinose in M9 medium. Cells were fixed at OD600 = 0.8. (A) Cell volume distributions of the cell populations were determined by Coulter cytometry. The *pflC* expression strain shows a mild cell division defect or delay. (B) BW25113 + pBAD24/*ybeM* and BW25113 + pBAD24/*ybiY* strains examined by phase-contrast microscopy. Expression of *ybeM* appears to cause a minor effect on cell length, compared to the control and *ybiY* expression strains. (PDF 1231 kb)

## Abbreviations

UPEC: Uropathogenic *E. coli*
UTI: Urinary Tract Infection
