# Supplementary material for: High-throughput sequencing of sorted expression libraries reveals inhibitors of bacterial cell division
Source: BMC Genomics. 2018 Oct 29;19:781. doi: 10.1186/s12864-018-5187-7 (PMC6206680; doi:10.1186/s12864-018-5187-7)
Supplement: Supplementary file 2 — Figure S1. Additional analyses of DNA sequencing data. (A) Plot showing the global read coverage of the UTI89 chromosome of the UTI89 genomic library, based on the read data from the “reference-unsorted 2” sample (Table 1). The library effectively covers the complete genome of UTI89, at a depth of 1–30 reads per bp. (B) A comparison of the number of identified enriched regions in replicate screens 1 and 2 by the MACS peak detection software (see Materials and Methods). The number of identified enriched regions were ranked by their significance score (−log10[P-value]), and the significance scores plotted against raw numbers of the identified regions. The dotted line represents the threshold of ≥70 P-value as the high-stringency criterion used to generate Table 2. (PDF 176 kb) [file 12864_2018_5187_MOESM2_ESM.pdf]

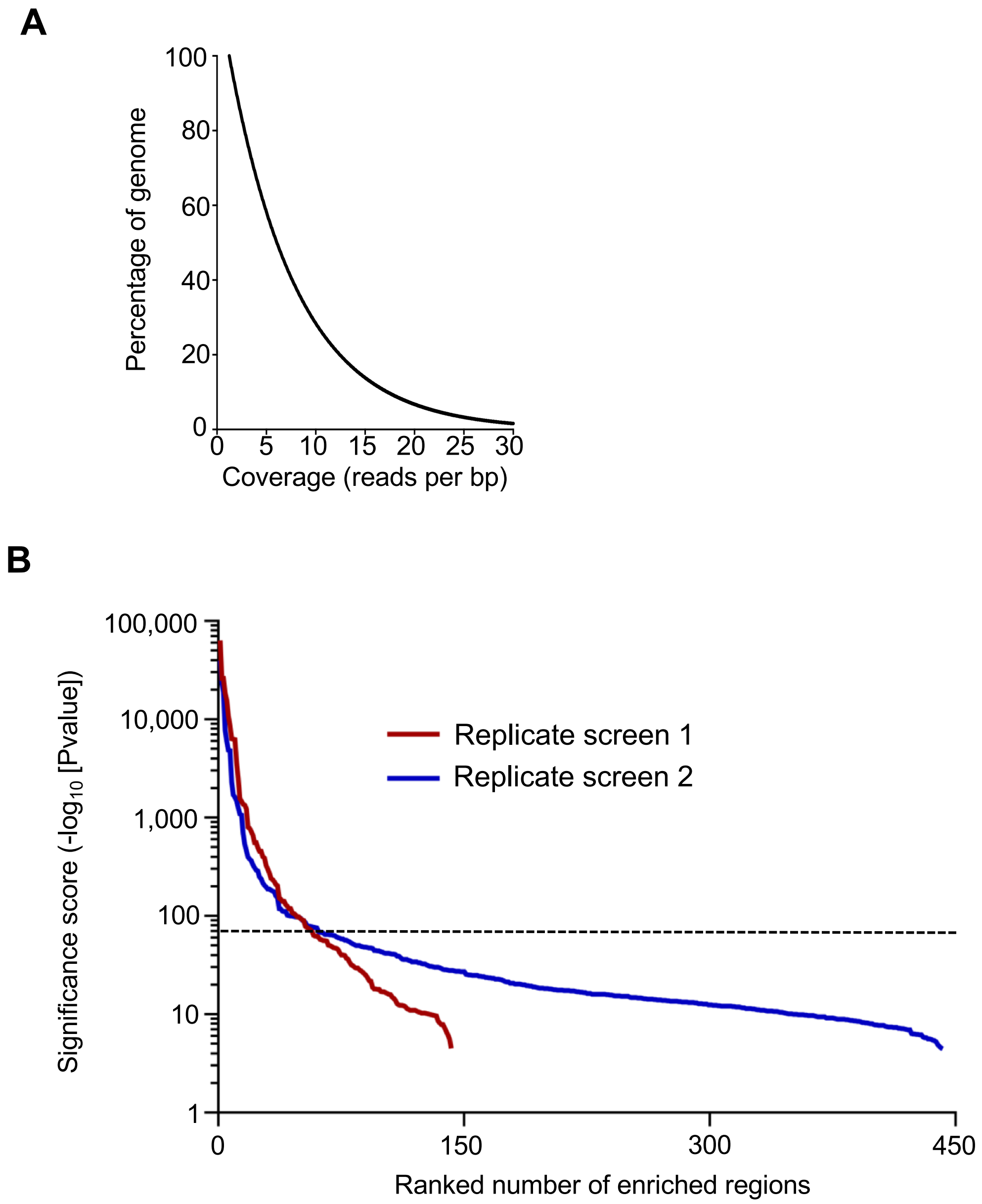

Supplementary Data Figure S1. Additional analyses of DNA sequencing data. (A) Plot showing the global read coverage of the UTI89 chromosome of the UTI89 genomic library, based on the read data from the "reference-unsorted 2" sample (**Table 1**). The library effectively covers the complete genome of UTI89, at a depth of 1-30 reads per bp. (B) A comparison of the number of identified enriched regions in replicate screens 1 and 2 by the MACS peak detection software (see *Materials and Methods*). The number of identified enriched regions were ranked by their significance score ($-\log_{10}$[P value]), and the significance scores plotted against raw numbers of the identified regions. The dotted line represents the threshold of ≥ 70 P-value as the high-stringency criterion used to generate **Table 2**.
