# Supplementary material for: High-throughput sequencing of sorted expression libraries reveals inhibitors of bacterial cell division
Source: BMC Genomics. 2018 Oct 29;19:781. doi: 10.1186/s12864-018-5187-7 (PMC6206680; doi:10.1186/s12864-018-5187-7)
Supplement: Supplementary file 4 — Figure S2. pptE over-expression causes major effects on E. coli cell structure and morphology. BW25113 + pBAD24/pptE was grown in M9 minimal medium and induced with L-arabinose at the indicated concentrations and then fixed and stained with Hoechst 33342 (DNA) and FM4–64 (membrane) stains and then visualised by fluorescence microscopy. (A) 0.2% L-arabinose. (B) 0.02% L-arabinose. The intracellular inclusion-like structures and FM4–64 staining abnormalities were observed at both induction concentrations, whereas significant filamentation was only observed at the higher L-arabinose concentration. (PDF 1184 kb) [file 12864_2018_5187_MOESM4_ESM.pdf]

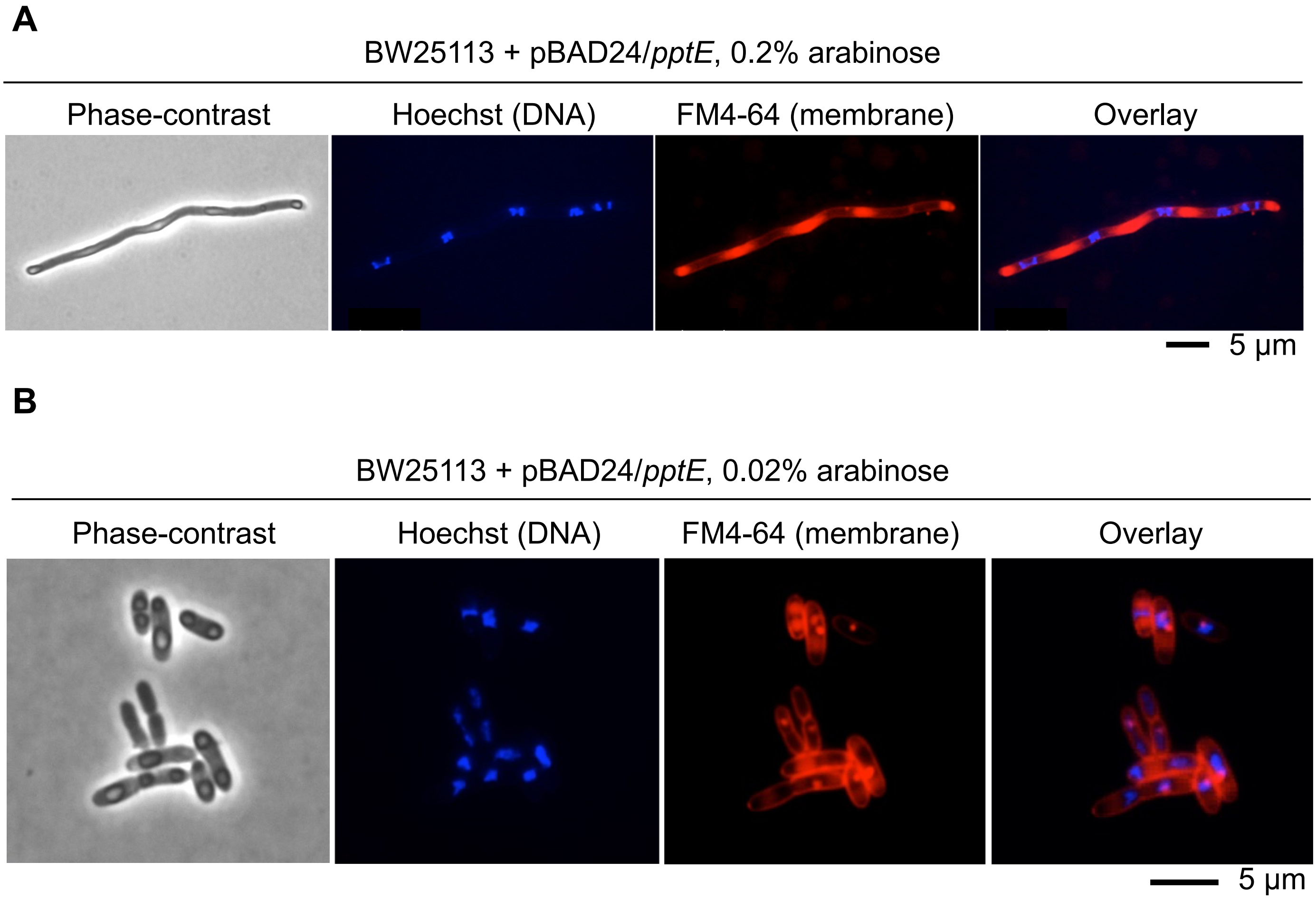

Supplementary Data Figure S2. *pptE* over-expression causes major effects on *E. coli* cell structure and morphology. BW25113+pBAD24/*pptE* was grown in M9 minimal medium and induced with L-arabinose at the indicated concentrations and then fixed and stained with Hoechst 33342 (DNA) and FM4-64 (membrane) stains and then visualised by fluorescence microscopy. (A) 0.2% L-arabinose. (B) 0.02% L-arabinose. The intracellular inclusion-like structures and FM4-64 staining abnormalities were observed at both induction concentrations, whereas significant filamentation was only observed at the higher L-arabinose concentration.
