# Supplementary material for: High-throughput sequencing of sorted expression libraries reveals inhibitors of bacterial cell division
Source: BMC Genomics. 2018 Oct 29;19:781. doi: 10.1186/s12864-018-5187-7 (PMC6206680; doi:10.1186/s12864-018-5187-7)
Supplement: Supplementary file 5 — Figure S3. ORFs fsaA, ybiY, aceE, lipA, sdaA, and pflC do not cause substantial filamentation when overexpressed. The ORFs were expressed from pBAD24 in BW25113 by induction with 0.2% L-arabinose in M9 medium. Cells were fixed at OD600 = 0.8. (A) Cell volume distributions of the cell populations were determined by Coulter cytometry. The pflC expression strain shows a mild cell division defect or delay. (B) BW25113 + pBAD24/ybeM and BW25113 + pBAD24/ybiY strains examined by phase-contrast microscopy. Expression of ybeM appears to cause a minor effect on cell length, compared to the control and ybiY expression strains. (PDF 1231 kb) [file 12864_2018_5187_MOESM5_ESM.pdf]

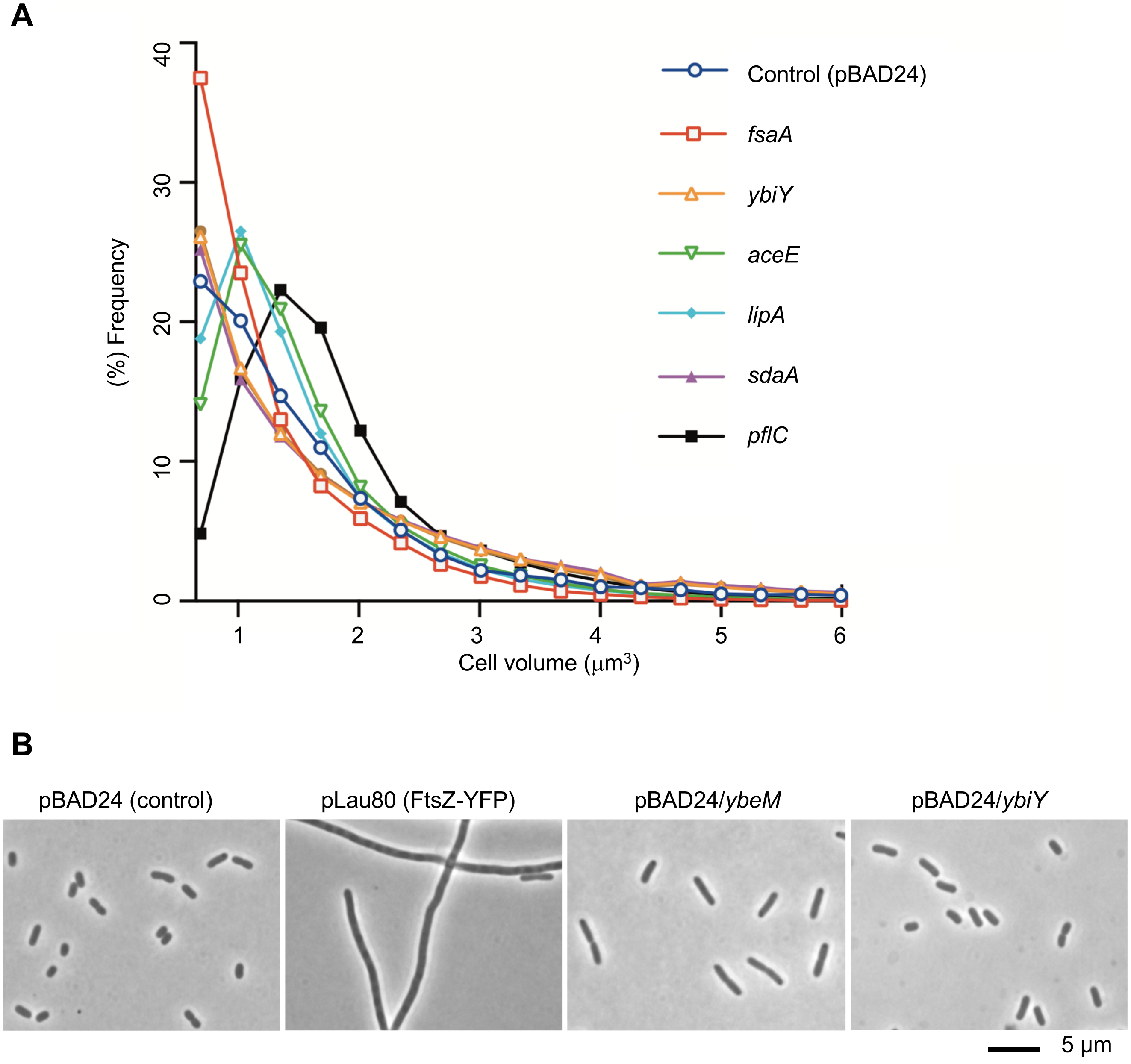

Supplementary Data Figure S3. ORFs *fsaA, ybiY, aceE, lipA, sdaA,* and *pflC* do not cause substantial filamentation when overexpressed. The ORFs were expressed from pBAD24 in BW25113 by induction with 0.2% L-arabinose in M9 medium. Cells were fixed at OD_{600} = 0.8. (A) Cell volume distributions of the cell populations were determined by Coulter cytometry. The *pflC* expression strain shows a mild cell division defect or delay. (B) BW25113 + pBAD24/*ybeM* and BW25113 + pBAD24/*ybiY* strains examined by phase-contrast microscopy. Expression of *ybeM* appears to cause a minor effect on cell length, compared to the control and *ybiY* expression strains.
